# Public Health in the United States.—I

**Published:** 1906-01-13

**Authors:** 


### PUBLIC HEALTH IN THE UNITED STATES.—I.

(*Continued from page* 243.)

### Control of Infectious Diseases.

This department of sanitary work constitutes the most important of all the duties, both of general and local boards of health. So far as State or general boards are concerned, the functions pertaining to this branch of work consist mainly in giving advice, in investigating the causes of outbreaks of disease, and in the circulation of general information among the people in regard to the methods of preventing the spread of disease. General boards are sometimes given co-ordinate powers with local boards, to be used in cases of emergency. The local board, on the other hand, in consequence of being (by reason of its mode of organisation) in closer touch with each individual member of the community, is usually clothed with extraordinary powers for the purpose of dealing with infectious diseases whenever and wherever they are observed among the people. The operations of local boards of health in this direction are enforced by the aid of laws, ordinances, and rules, and the different methods employed are (1) notification, (2) isolation, and (3) disinfection. To this may be added, for the purpose of securing immunity from certain diseases, the provision of vaccination as a protection against the dangers of small-pox, and of certain anti-toxins for securing immunity against other diseases, notably against diphtheria. By far the greater use, however, of these latter products has been of a therapeutic nature in treating disease when actually existing in this individual. But custom has in this case so closely associated the methods of prevention with those of treatment as to place the distribution of such therapeutic remedies largely within the control of the sanitary authority.

*Notification.*—Till within the past twenty-five years notification of infectious disease was scarcely recognised in the States except in the single instance of small-pox. It is now quite generally the custom throughout all the more densely settled States, and specially in the cities and large towns, to require notification of small-pox, diphtheria (including membranous croup), scarlet fever, and typhoid fever. To these may be added, in a more limited degree, measles, cerebro-spinal meningitis, yellow fever, leprosy, Asiatic cholera whooping-cough, and German measles. The growth of this important measure has been much more rapid in the United States in the past fifteen years than in all other years preceding. Notwithstanding the general recognition of the infectious character of tuberculosis, the propriety of requiring its notification in common with other infectious diseases, and on similar terms, does not appear to have become generally acknowledged in the United States, and the only large cit in which such notification is compulsory is New York, where by the following order of the Board of Health of the city, such notice is called for:—

Section 225 of the Sanitary Code, adopted January 19th, 1897, classes pulmonary tuberculosis as "an infectious and communicable disease." Under the provisions of this section physicians are required to report to the health department the name, address, age, sex, and occupation of every case of pulmonary tuberculosis coming under their professional care. The information thus received is solely for registration, and cases so reported are not visited by the inspectors of this department, nor are they interfered with in any way, except upon the request of the attending physician. The residences of all cases of tuberculosis reported to this department are visited by medical inspectors, who then give information with regard to the nature of the disease and the precautions necessary to prevent the infection of others. When residences occupied by consumptives are vacated through the death or removal of the patient, the inspectors recommend the renovation required to free them from infection. The orders for such renovation are enforced by the Board of Health.

*Isolation.*—The practice of requiring the separation from the community at large of persons suffering with small-pox has been recognised for many years, and those attacked with yellow fever have been dealt with in the same manner in later years, but it has not been till the last 25 years that the laws of the States have definitely provided for the separation of those who are affected with other infectious diseases. For the purpose of facilitating the work of isolation and of preventing the access of persons from outside the dwelling, placards or flags are commonly attached to the house in some conspicuous place, in order to notify the community of the existence of infection. Except in the case of small-pox, it is commonly the custom to permit the wage-earners of the family to continue their occupations, under proper restrictions as to disinfection, bathing, and change of clothing. It is now commonly the practice to require the isolation, either in their own dwellings or in hospitals provided for the purpose, of persons suffering with small-pox, diphtheria, and scarlet fever, and also the exclusion from school of scholars suffering with the same diseases, and of other scholars living in the same family with pupils so suffering. Isolation is also applied to cases of measles, and also in a less rigid manner to typhoid fever and whooping-cough.

*Isolation Hospitals.*—The practice of providing special hospitals for the treatment of persons suffering with infectious diseases has not become so widespread in the United States as it has in Great Britain. Pest-houses and small-pox hospitals have, however, existed from quite an early period, but on account of the extremely irregular occurrence of these diseases, and of the need of keeping them closed much of the time, their equipment has usually been of a primitive character. It is only within the past 10 or 15 years that cities have begun to recognise the need of special provision for this class of diseases. With reference to the isolation of persons suffering with tuberculosis, a similar want of adequate provision by public authority is manifest, but a general awakening on this subject is now taking place. Provision has been made in general States, mostly by private authorities.

*Disinfection.* — The methods employed for disinfection after the occurrence of infectious diseases, in the United States, have been very much the same as those in use in the different countries of Europe, specially since the very important investigations of Professor Koch, upon the value of the different substances used for disinfection, were made public. Apparatus for steam disinfection has been introduced in the largest cities for the disinfection of movable material, and similar apparatus is in use in connection with many public institutions, isolation hospitals, and also at the different quarantine stations in seaport cities. Within the past seven years or more the use of formaldehyde, by means of various forms of apparatus, has very largely superseded the use of sulphurous acid for the disinfection of closed apartments.

*Vaccination.*—Laws relating to vaccination were first enacted during the first decade of the nineteenth century, and have been followed by various amendments and limitations from that time till the present. One of the most efficient laws relating to this subject, is that which exists in many of the States requiring that unvaccinated children shall not be admitted to the public schools. While there are compulsory laws in some States, it cannot be said with truth that such laws are thoroughly enforced, to the extent of securing the vaccination of the entire population over two years of age, as is done, for example, throughout the German empire. The vaccinated portion of the inhabitants of the United States may be estimated at not far from 90 per cent. of the whole, and the revaccinated portion at probably 50 per cent.

